# 3-Dimensional Echocardiography and 2-D Strain Analysis of Left Ventricular, Left Atrial and Right Ventricular Function in Healthy Brazilian Volunteers

**DOI:** 10.5935/abc.20190155

**Published:** 2019-11

**Authors:** Roberto M. Saraiva, Eliza Maria B. Scolin, Nicole P. Pacheco, Maria Eduarda Bouret, Mauro Felippe Felix Mediano, Marcelo T. Holanda, Andréa R. da Costa

**Affiliations:** 1 Instituto Nacional de Infectologia Evandro Chagas, Fundação Oswaldo Cruz, Rio de Janeiro, RJ - Brasil; 2 Departamento de pesquisa e Educação, Instituto Nacional de Cardiologia, Rio de Janeiro, RJ - Brasil

**Keywords:** Cardiovascular Diseases, Echocardiography, Three-Dimensional, Reference Values, Left Ventricular Function, Right Ventricular Function, Strain, Speckle Tracking

## Abstract

**Background:**

New echocardiographic techniques are used in the diagnosis and prognosis of many heart diseases. However, reference values in different populations are still needed for several of these new indexes. We studied these new echocardiographic parameters in a group of Brazilians with no known cardiovascular disease.

**Objective:**

To study values for new echocardiographic indexes in Brazilians without known cardiovascular disease and their correlation with age.

**Methods:**

Cross-sectional study that included healthy individuals who underwent three-dimensional echocardiography (3DE) and two-dimensional speckle tracking echocardiography (STE) strain (e) analysis. Left atrial (LA) and left ventricular (LV) function were analyzed by 3DE and STE, and right ventricular (RV) function by STE. P values < 0.05 were considered significant.

**Results:**

Seventy-seven subjects (46.7% men; 40.4 ± 10.4 years) were included. Maximum, minimum and pre-atrial contraction (pre-A) LA volumes (ml/m^2^) were 21.2 ± 5.5, 7.8 ± 2.5, and 11.0 ± 3.1, respectively. Peak positive global LA e (LAScd), peak negative global LA e and total global LA e (LASr) were 17.4 ± 5.2%, -13.2 ± 2.0% and 30.5 ± 5.9%, respectively. LV end-diastolic and end-systolic volumes (ml/m^2^) measured 57 ± 12 and 24 ± 6, and 3D LV ejection fraction measured 58 ± 6%. Global LV longitudinal, circumferential and radial e were -19 ± 2%, -19 ± 3%, and 46 ± 12%, respectively. LV torsion measured 1.6 ± 0.7^0^ /cm. Global longitudinal RV e (RV-GLS) and RV free wall strain were -22 ± 3% and -24 ± 5%. Minimum LA and pre-A volumes, LV apical rotation, torsion and RV-GLS increased with age, while total and passive LA emptying fractions, LAScd, LASr, LV end-diastolic and end-systolic volumes decreased with age.

**Conclusion:**

Values for new echocardiographic indexes in Brazilians without known cardiovascular disease and their correlation with age are presented.

## Introduction

New echocardiographic techniques are being increasingly used in the diagnosis and prognosis of many cardiac diseases.^1-3^ However, reference values in different populations are still needed for several of the new indexes derived from those techniques. Left ventricular (LV), left atrial (LA) and right ventricular (RV) function can be evaluated by both three-dimensional echocardiography (3DE) and two-dimensional speckle tracking echocardiography (STE) strain (e) analysis. Reference values for some of the indexes derived from those techniques can be found in recent guidelines but are still missing in many of them.^1^ Consequently, studies regarding the evaluation of LV, LA, and RV function in different conditions require control groups, which may not represent a real consistent sample of a normal population. Furthermore, the Brazilian population is often underrepresented in studies that assessed reference values for those parameters. Therefore, we studied new echocardiographic parameters and their correlation with age in Brazilians without known cardiovascular disease.

## Methods

### Study subjects

This is a cross-sectional prospective study that recruited subjects among those referred to our institution, who tested negative for Chagas disease in two different serological tests, had no known diseases, presented normal physical examination, normal electrocardiogram and echocardiograms with normal global and segmental LV systolic function and no significant valvular heart disease. Race/ethnicity was obtained by self-description. *The study was conducted at Instituto Nacional de Infectologia Evandro Chagas* (INI), from Fundação Oswaldo Cruz, located in the city of Rio de Janeiro, Brazil*. INI is a public institution dedicated to clinical research on infectious diseases and a reference center for diagnosis and treatment of Chagas’ disease.*

This article is derived from our project “Analysis of the cardiac performance by new echocardiographic techniques in patients with Chagas disease”.^4,5^ We used a convenience sample.

### Echocardiography

Studies were performed using phased-array ultrasound system (Vivid 7, GE Medical Systems, Milwaukee, WI) equipped with M4S phased-array and 1.5- to 4-MHz 4 matrix-array transducers. Cardiac dimensions measured by M-mode and 2D echocardiography and Doppler measurements were obtained as recommended.^1,6^ Echocardiograms were reviewed offline and two-dimensional e and 3DE analyses were performed with software Echopac PC workstation version 108.1.12 (GE Medical Systems) with a 4D LV volume analysis plug-in (Tomtec Imaging Systems Gmbh). The same software designed to calculate LV e was used to analyze RV and LA e. All 2D clips analyzed were acquired at high frame rate (>60 frames/s). Studies were acquired by one echocardiographer and analyzed by two echocardiographers.

### Two-dimensional strain analysis

#### LA strain analysis

LA e was determined as previously described^7^ using apical 4- and 2-chamber views. P-wave onset was used as the reference point, which enabled the recognition of peak positive global LAe (LAScd), which corresponded to LA conduit function, peak negative global LAe (LASct), which corresponded to LA contractile function, and the sum of those previous values (LASr), which corresponded to LA reservoir function.

#### Two-dimensional LV and RV strain analysis

LV global longitudinal e (LV-GLS), LV global circumferential e (LV-GCS) and LV global radial e (LV-GRS) were calculated as previously described.^4,5^ LV-GCS and LV-GRS were the average of the peak average for LV-GCS and LV-GRS obtained at short-axis views at the basal, mid and apical levels. LV-GLS was the average of the peak average for LV-GLS obtained at 4-, 2- and 3-chamber views. If tracking quality was not good in two segments of the same acoustic window, that view was excluded from global LV e calculation.

RV longitudinal e (RV-GLS) was calculated using focused 4-chamber apical view. RV free wall e (RV-fwLS) was calculated as the average of the basal, mid and apical RV free wall segments.

#### Left ventricular torsion calculation

LV torsion and twist were calculated as previously described.^5^ LV twist was defined as the net difference of LV rotation (LVrot) between apical and basal short-axis planes obtained from STE analysis and LV torsion as the LV twist divided by the end-diastolic LV longitudinal length. Counterclockwise LVrot as viewed from apex was expressed as a positive value.

### LA and LV volume and function analyses by 3DE

3DE 4-beat full volume images were acquired during breath hold in end expiration from apical views. Volume rate varied from 17 to 25 volumes/s.

LA 3D images were analyzed as previously described.^5^ The software showed maximum and minimum LA volume and pre-contraction LA volume was obtained from analysis of time-volume curves. Total LA emptying fraction was calculated as [(maximum LA volume - minimum LA volume)/maximum LA volume] x 100. Active LA emptying fraction was calculated as [(pre-contraction LA volume - minimum LA volume)/pre-contraction LA volume] x 100. Passive LA emptying fraction was calculated as [(maximum LA volume - pre-contraction LA volume)/maximum LA volume] x 100.

LV volume and 3D LV ejection fraction were measured using a similar approach.^5^

### Statistical analysis

Calculations were done using the software package MedCalc 12.5.0.0. Continuous variables were expressed as mean ± standard deviation and discrete variables as absolute and percentage values. Echocardiographic variables passed standard tests of normality (Kolmogorov-Smirnov test). Normally distributed data were compared by unpaired Student’s t-tests. Discrete variables were compared by contingency tables. Correlation between age and echocardiographic parameters was tested by Pearson’s correlation and classified according to Zegers et al.^8^ Stepwise multiple regression analysis was used to analyze independent correlation between echocardiographic indexes and age, gender, body mass index (BMI) and ethnicity. Interobserver and intraobserver agreements were determined after offline re-analysis of recorded clips of 15 randomly selected subjects and assessed by Bland-Altman analysis. P values < 0.05 were considered significant.

## Results

### Participants

**Figure 1:**
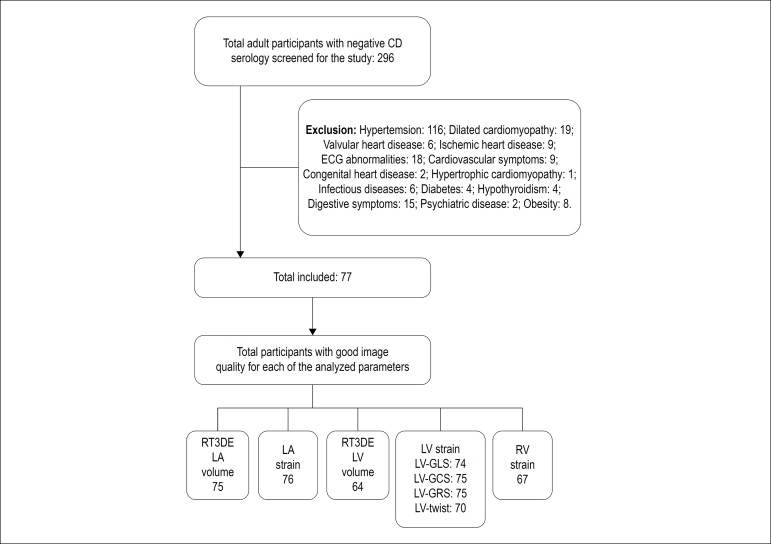
Flowchart of enrollment of subjects in the present study.

A total of 296 adult subjects with negative Chagas disease serology were screened from March 2010 to November 2013, and from May 2016 to July 2017. After applying exclusion criteria, 77 subjects (36 men; 46.7%) were included (Figure 1). Regarding age groups, 39 participants were 18 to 39 years old, 35 participants were 40 to 60 years old and 3 participants were 60 years old or older. Most subjects were born in the Southeast and Northeast of Brazil and were white. Distribution by state was as follows: 24 were born in Rio de Janeiro, 7 in Minas Gerais, 1 in Espírito Santo, 12 in Bahia, 9 in Paraíba, 8 in Ceará, 5 in Pernambuco, 5 in Alagoas, 1 in Piauí, 1 in Sergipe and 4 in Pará. There was no significant difference in age, BMI, place of origin and ethnicity distribution between men or women (Table 1).

**Table 1:** Characteristics of study participants

	All n = 77		Male n = 36		Female n = 41	P value^a^
Age, years	40.4 ± 10.4 (range 19–78)		39.5 ± 9.7 (range 19–62)		41.3 ± 11.0 (range 21–78)	0.45
BMI, kg/m^2^	25.0 ± 3.2		25.1 ± 3.0		24.9 ± 3.3	0.78
SBP, mmHg	122 ± 12		124 ± 10		121 ± 13	0.28
DBP, mmHg	76 ± 8		77 ± 7		75 ± 9	0.49
**Place of origin**						0.15
Southeast	32 (41.5%)		19 (52.7 %)		13 (31.7%)	
Northeast	41 (53.2%)		16 (44.4%)		25 (61%)	
North	4 (5.2%)		1 (2.8%)		3 (7.3%)	
**Ethnicity**						0.13
White	42 (54.5%)		24 (66.6%)		18 (43.9%)	
Afro-Brazilian	11 (14.3%)		4 (11.1%)		7 (17.1%)	
Mixed/Browned skinned	24 (31.1%)		8 (22.2%)		16 (39.0%)	
**2D echocardiogram**						
LA, cm	3.4 ± 0.4		3.5 ± 0.5		3.2 ± 0.3	0.009
LVd, cm	5.0 ± 0.5		5.3 ± 0.4		4.7 ± 0.4	< 0.0001
LVs, cm	3.0 ± 0.4		3.2 ± 0.4		2.8 ± 0.4	< 0.0001
LV ejection fraction, %	68 ± 6		67 ± 5		69 ± 6	0.10
LV S’, cm/s	9.6 ± 2.0		9.8 ± 2.0		9.4 ± 2.0	0.32
LV mass, g/m^2^	60 ± 12		63 ± 13		58 ± 11	0.07
E/A ratio	1.6 ± 0.5		1.7 ± 0.5		1.6 ± 0.4	0.08
DT, ms	160 ± 34		161 ± 34		160 ± 35	0.81
E’, cm/s	13.6 ± 2.9		14.1 ± 2.4		13.3 ± 3.2	0.22
E/E’ ratio	6.2 ± 1.8		5.5 ± 1.0		6.9 ± 2.0	0.0003
Vp, cm/s	73 ± 23		72 ± 26		74 ± 120	0.67
E/Vp	1.2 ± 0.4		1.1 ± 0.3		1.3 ± 0.4	0.21
RV S’, cm/s	14.6 ± 2.3		14.7 ± 2.4		14.6 ± 2.2	0.93
TAPSE, mm	24 ± 4		25 ± 4		24 ± 3	0.71
RVSP, mmHg	26 ± 5		26 ± 5		25 ± 5	0.45

A: peak late wave diastolic filling velocity; BMI: body mass index; DBP : diastolic blood pressure; DT: E-wave deceleration time; E: peak early wave diastolic filling velocity; E’: peak early diastolic mitral annulus velocity; LA: left atrium; LV: left ventricle; LVd: LV end-diastolic diameter; LVs: LV end-systolic diameter; RV: right ventricle; RVSP: RV systolic pressure; S’: peak systolic mitral annulus velocity; SBP: systolic blood pressure; TAPSE: tricuspid annular plane systolic excursion; Vp: propagation velocity.

a Student’s t-test or contingency tables comparing men vs. women, as appropriate. n (%); mean ± SD.

Men presented larger LA and LV diameters and a tendency for larger LV mass while E/E’ ratio was higher in women (Table 1).

### 3D echocardiography analysis of LA volume, function and strain

Except for two, good image quality was obtainable for 3DE LA volume analysis from all participants. Except for one, LA strain analysis was feasible in all participants.

LA volume and function values obtained by 3DE and STE are presented in Table 2. Pre-A LA volume was slightly larger and passive LA emptying fraction was smaller in men than in women. There were no differences regarding LA e parameters between men and women (Table 2).

**Table 2:** 3D echocardiographic LA volume and function and LA strain analyses

	All n = 77	Men n = 36	Women n = 41	P value^a^
**3DE**				
Max LA Vol, mL/m^2^	21.2 ± 5.5	21.6 ± 5.5	20.9 ± 5.5	0.63
Min LA Vol, mL/m^2^	7.8 ± 2.5	8.3 ± 2.6	7.4 ± 2.4	0.11
Pre-A LA Vol, mL/m^2^	11.0 ± 3.1	11.8 ± 3.1	10.4 ± 3.0	0.04
Total LA EF, %	63 ± 8	61 ± 7	64 ± 8	0.08
Active LA EF, %	29 ± 9	29 ± 9	29 ± 9	0.74
Passive LA EF, %	48 ± 10	45 ± 8	50 ± 11	0.03
**LA Strain**				
LASct, %	-13.2 ± 2.0	-13.1 ± 2.4	-13.2 ± 1.5	0.70
LAScd, %	17.4 ± 5.2	16.8 ± 5.0	17.8 ± 5.3	0.36
LASr, %	30.5 ± 5.9	29.8 ± 6.0	31.1 ± 5.8	0.35

EF: emptying fraction; LA: left atrium; LASct: peak negative global LA ε; LAScd: peak positive global LA ε; LASr: total global LA ε; Max: maximum; Min: minimum; Pre-A LA vol: LA volume at the onset of LA contraction; 3DE: three-dimensional echocardiography; Vol: volume.

a Student’s test-t comparing men vs. women.

### 3D echocardiography analysis of LV volume, function and strain

Images of enough quality to analyze 3D LV volumes were obtained in 64 (83%) participants. Three patients were excluded from LV-GLS analysis and two patients were excluded from LV-GCS and LV-GRS analyses due to poor imaging quality. Seven patients were excluded from LV twist and torsion analyses due to poor imaging quality for LVrot analysis in either basal or apical short-axis views.

Values for LV volume and function obtained by 3DE and STE are presented in Table 3. LV end-diastolic volume was larger and end-systolic volume presented a tendency to be larger in men than women while 3D LV ejection fraction did not differ between them. LV-GCS was slightly higher in women than in men, while LV-GLS, LV-GRS, LV rotation and torsion did not differ between them (Table 3).

**Table 3:** 3D echocardiographic analysis of LV volume, function and strain

	All n = 77	Men n = 36	Women n = 41	P value^a^
**3DE**				
LV end-diastolic volume, mL/m^2^	57 ± 12	60 ± 10	54 ± 13	0.04
LV end-systolic volume, mL/m^2^	24 ± 6	25 ± 5	22 ± 7	0.05
Ejection fraction, %	58 ± 6	58 ± 6	58 ± 6	0.61
**LV Strain**				
LV-GLS, %	-19 ± 2	-19 ± 2	-20 ± 2	0.06
LV-GCS, %	-19 ± 3	-18 ± 3	-20 ± 3	0.03
LV-GRS, %	46 ± 12	44 ± 12	48 ± 12	0.19
Peak apical rotation, ^0^	8.7 ± 4.2	7.9 ± 4.1	9.3 ± 4.2	0.19
Peak basal rotation, ^0^	-5.6 ± 3.0	-5.2 ± 2.5	-5.9 ± 3.3	0.31
Peak twist, ^0^	13.5 ± 5.0	12.6 ± 4.4	14.2 ± 5.4	0.18
Peak torsion, ^0^/cm	1.6 ± 0.7	1.4 ± 0.5	1.7 ± 0.7	0.07
Untwist,^0^/s	-116 ± 32	-110 ± 34	-119 ± 31	0.26

LV: left ventricle; LV-GLS: global LV longitudinal strain; LV-GCS: global LV circumferential strain; LV-GRS: global LV radial strain; 3DE: three-dimensional echocardiography.

a Student’s test-t comparing men vs. women.

### RV strain analysis

Good imaging quality for RV e analysis was obtainable from 67 (87%) participants.

RV function values obtained by STE are presented in Table 4. RV e parameters did not differ between men and women (Table 4).

**Table 4:** RV strain analysis

	All n = 77	Men n = 36		Women n = 41	P value^a^
RV-GLS, %	-22 ± 3	-22 ± 3		-22 ± 3	0.86
RV-fwLS, %	-24 ± 5	-25 ± 5		-24 ± 4	0.17

RV: right ventricle; RV-fwLS: RV free wall longitudinal strain; RV-GLS: global RV longitudinal strain.

a Student’s test-t comparing men vs. women.

### Correlation between age and new echocardiographic indexes

**Figure 2:**
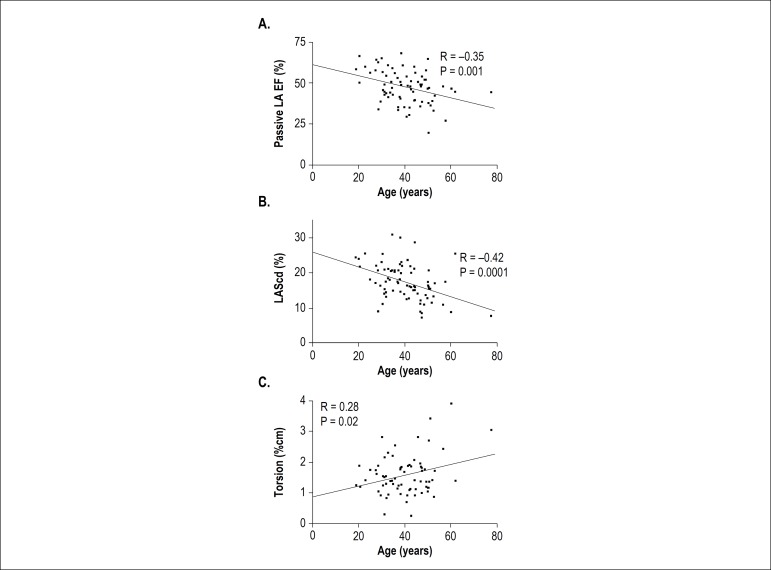
Correlation between age and passive LA EF (A), LAScd (B), and LV torsion (C). LAScd: peak positive global LA ε; LA EF: left atrial emptying fraction; LV: left ventricle.

Regarding LA volume and function, age presented fair positive correlation with minimum LA (r = 0.27, p = 0.02) and pre-A volume (r = 0.33, p = 0.004), and fair negative correlation with total LA emptying fraction (r = -0.26, p = 0.02) and passive LA emptying fraction (r = -0.35, p = 0.001, Fig. 2A). Age presented a moderate negative correlation with LAScd (r = -0.42, P=0.0001, Fig. 2B) and a fair negative correlation with LASr (r = -0.39, p = 0.0004). Age did not correlate with maximum LA volume, active LA emptying fraction and LASct.

Regarding LV volume and function, age presented fair negative correlation with LV end-diastolic (r = -0.31, p = 0.01) and end-systolic volume (r = -0.34, p = 0.007), and fair positive correlation with apical LV rotation (r = 0.29, p = 0.02), twist (r = 0.28, p = 0.02) and torsion (r = 0.28, p = 0.02, Fig. 2C), but no significant correlation with LV ejection fraction, LV-GLS, LV-GCS or LV-GRS.

RV-GLS presented fair positive correlation with age (r = 0.31; p = 0.01) while RV-fwLS did not correlate with age.

Among men, age presented a fair negative correlation with passive LA emptying fraction (r = -0.31, p = 0.07). Other variables did not reach statistical significance. Among women, age presented moderate positive correlation with minimum LA (r = 0.51, p = 0.0006) and pre-A volume (r = 0.53, p = 0.0003), fair negative correlation with total LA emptying fraction (r = -0.41, p = 0.007), and moderate correlation with passive LA emptying fraction (r = -0.43, p = 0.005). Among women, age also presented moderate negative correlation with LAScd (r = -0.59, p < 0.0001) and LASr (r = -0.58, p < 0.0001), fair negative correlation with LV end-diastolic (r = -0.34, p = 0.04) and end-systolic volume (r = -0.39, p = 0.02), and moderate positive correlation with apical LV rotation (r = 0.46, p = 0.003), twist (r = 0.43, p = 0.006) and torsion (r = 0.42, p = 0.007), and fair positive correlation with LV-GLS (r = 0.34, p = 0.03) and RV-GLS (r = 0.32, p = 0.05).

We analyzed whether the correlations between age and new echocardiographic indexes were independent from gender, BMI and ethnicity (Table 5). The analyzed variables accounted for only a modest percentage of the variability of the new echocardiographic indexes. Minimum LA volume correlated with age. LA pre-A volume and total LA emptying fraction correlated with age and gender. Passive LA emptying fraction correlated with age, gender and BMI. LASr and LAScd correlated with age and BMI. LV end-diastolic and end-systolic volumes correlated with age. LV-GCS correlated with gender. LV-GLS correlated with BMI. Age correlated with torsion and apical rotation. RV-GLS correlated with BMI and ethnicity and RV-fwGLS correlated with BMI.

**Table 5:** Multiple linear regression to determine independent predictors of new echocardiographic indexes

	β coefficients ± standard error	P value	adjusted R^2^
**Min LA Vol**			
Age	0.07 ± 0.03	0.02	0.06
**Pre-A LA Vol**			
Age	0.10 ± 0.03	0.002	0.15
Male	1.57 ± 0.7	0.02	
**Total LA EF**			
Age	-0.21 ± 0.09	0.02	0.09
Male	-3.7 ± 1.8	0.04	
**Passive LA EF**			
Age	-0.27 ± 0.11	0.01	0.24
Male	-5.3 ± 2.0	0.01	
BMI	-0.9 ± 0.3	0.01	
**LAScd**			
Age	-0.16 ± 0.05	0.003	0.29
BMI	-0.60 ± 0.17	0.0006	
**LASr**			
Age	-0.17 ± 0.06	0.0007	0.25
BMI	-0.67 ± 0.20	0.001	
**LV end-diastolic volume**			
Age	-0.36 ± 0.14	0.01	0.08
**LV end-systolic volume**			
Age	-0.19 ± 0.07	0.007	0.10
**LV-GLS**			
BMI	0.23 ± 0.09	0.01	0.07
**LV-GCS**			
Male	1.6 ± 0.7	0.03	0.05
**Peak apical rotation**			
Age	0.12 ± 0.05	0.01	0.07
**Peak twist**			
Age	0.14 ± 0.06	0.02	0.08
**Peak torsion**			
Age	0.02 ± 0.007	0.02	0.07
**RV-GLS**			
BMI	0.31 ± 0.10	0.002	0.18
Ethnicity	0.71 ± 0.10	0.05	
**RV-fwLS**			
BMI	0.56 ± 0.17	0.002	0.13

EF: emptying fraction; LA: left atrium; LASct: peak negative global LA ε; LAScd: peak positive global LA ε; LASr: total global LA ε; Max: maximum; Min: minimum; Pre-A LA vol: LA volume at the onset of LA contraction; 3DE: three-dimensional echocardiography; Vol: volume; RV-fwLS: RV free wall longitudinal strain; RV-GLS: global RV longitudinal strain. ^a^ Student’s test-t comparing men vs. women.

### Intra- and interobserver variabilities

The intra- and interobserver variabilities for 3DE LA volumes, LA and LV e of our group using the same machine used in this article have already been published.^4,5^

The mean differences (±1.96 SD) for intraobserver agreement for 3DE LV volumes were -2.4 ml/m^2^ (±5.0 ml/m^2^), -1.5 ml/m^2^ (±4.0 ml/m^2^) for end-diastolic and end-systolic LV volumes, respectively, and 0.5% (±6.7%) for LV ejection fraction. The mean differences for interobserver agreement for 3DE LV volumes were -7.8 ml/m^2^ (±11.0 ml/m^2^), -2.4 ml/m^2^ (±5.6 ml/m^2^) for end-diastolic and end-systolic LV volumes, respectively, and -1.5% (±7.2%) for LV ejection fraction.

The mean differences for intraobserver agreement for RV e were -0.3% (±2.2%) and -0.1% (±4.0%) for RV-GLS and RV-fwGLS, respectively. The mean differences for interobserver agreement for RV e were -0.4% (±2.8%) and 0.1% (±5.6%) for RV-GLS and RV-fwGLS, respectively.

## Discussion

In this article, we present values for new echocardiographic indexes obtained from Brazilians without known cardiovascular diseases. Most subjects were born in Southeastern and Northeastern Brazil. Our study included a population of both sexes made up, in its majority, of white people, followed by mixed/brown skinned and Afro-Brazilians, similar to the ethnic distribution described by the IBGE demographic census of 2010: 44.7% of white people, 43.1% of mixed/brown skinned, 7.6% of Afro-Brazilians, 1.1% of Asian-Brazilians and 0.43% of native Indians (https://www.ibge.gov.br/estatisticas-novoportal/sociais/populacao/9662-censo-demografico-2010.html?edicao=10503&t=resultados).

LA and LV diameters measured by 2D-echocardiography were larger in men and LV mass also tended to be larger in men, as those parameters are gender-dependent.^1^ Other studies with Brazilians also found the differences described in this paper between men and women for LA and LV diameters and LV mass.^9,10^ We also found that E/E’ ratio was higher in women, as described by others.^11,12^ Therefore, gender-specific reference values for Doppler measures may be needed in clinical practice.

Regarding LA volumes, we found indexed mean values within the normal range published elsewhere.^13,14^ Males presented minimum LA volume slightly larger than women, which is in accordance with previous studies,^13^ but not with others.^14^

The average values for LAScd and LAsr described by us are within the range described for LA e reference values,^15^ while LASct described by us was lower than previously reported.^15^ However, there is a wide variation between studies that described LA e reference values due to methodological variations (reference frame set to zero strain: P wave or QRS complex onset; inclusion or exclusion of LA roof; apical views used for LA e analysis; machines or software packages from different vendors),^15-17^ and influence of age,^7^ LV diastolic function,^7^ LV end-diastolic pressure,^18^ and image quality over LA e parameters.

Ageing correlated with worsening of LA reservoir and conduit function as demonstrated by positive correlation with minimum LA and pre-A volumes and negative correlation with total and passive LA emptying fractions, LAScd, and LASr. Others also found that age correlated positively with minimum LA volume and negatively with total LA emptying fraction^13^ or that all LA volumes increased with age.^14^ Others also found that LAScd, and LASr decreased with age.^7^ The correlation found with age may be partly explained by the known effect of age on LV diastolic function. On multiple regression analysis, age remained the most common covariate associated with 3DE and LA e parameters. Male gender was also positively associated with LA pre-A volume and negatively associated with total and passive LA emptying fractions. Others found higher maximum and minimum 3DE LA volumes in men,^13^ while others found no gender influence on 3D LA volumes.^14^ Passive LA emptying fraction, LASr, and LAScd decreased with BMI, demonstrating a possible effect of overweight on LA reservoir and conduit function. In fact, others found that total and passive LA emptying fractions and LA e decreased with BMI.^19^

The 3DE LV volumes described by us are similar to those described previously,^20,21^ but larger than those described by others.^22,23^ These differences may be attributed to different ethnic backgrounds, as the first two studies are composed of white patients and the last two, patients with different backgrounds. The 3D LV ejection fraction described by us is similar to the one described in those articles. We found larger LV volumes in men, as in others.^20,21^ However, after multiple regression analysis, only age was related to 3D LV volumes. We described a negative correlation between age and LV volumes, as previously described.^21,22^

The global LV e found in our population did not differ from previous studies that analyzed LV-GLS,^24-28^ LV-GCS^25,26,28^ and LV-GRS.^25-28^ However, other studies described higher LV-GCS in normal subjects than us^27^ and a recent meta-analysis of 24 studies found mean LV-GLS and LV-GRS values very similar to our findings, but higher mean LV-GCS than we did.^29^ We found that LV-GCS was higher in women and a tendency for LV-GLS to be higher in women, which is consistent with others that found higher LV-GCS^25,27^ and LV-GLS^26,27^ in women. Multiple regression analysis confirmed the positive independent relationship between women and absolute LV-GCS values. Although others found correlation between LV e and age,^25,28^ we could not confirm those results. Kocabay et al.^26^ did not find any difference in LV e between different age groups either.^26^ Multiple regression analysis demonstrated that LV-GLS absolute values decreased with BMI, as previously described.^26^

The LV rotation and twist described by us are similar to the ones previously described,^30^ but lower than the ones described by others,^26,31^ and higher than the ones described elsewhere.^28^ LV twist and torsion increased with age, as described by others.^26,28^ Reasons for differences found in mean LV e and torsion between our study and others may be due to heterogeneity introduced by different machine vendors,^32^ age,^25^ gender distribution^25-27^ and the technique used to measure LV-GLS and LV-GRS.^27^

Regarding RV strain, the RV-fwLS described by us was similar to that found in previous studies,^33^ but slightly lower than that described by others.^34^ Although we did not find correlation between RV e and age after multiple regression analysis or gender-based differences, others^33^ found that RV-fwLS decreased with ageing, and was lower among men. After multiple regression analysis, RV-GLS absolute values decreased with BMI and ethnicity and RV-fwGLS also decreased with BMI. Although we cannot compare these results to the ones found in the literature, LV-GLS is described to decrease with BMI^26^ and ethnicity may influence LV e.^25^ More studies in subjects without known diseases are needed to validate reference values.

### Limitations

Our data was obtained using a machine from a single vendor but the technology of 3DE measurements and STE may differ between different vendors and Echopac software versions. In fact, LV-GLS measured with seven different brands presented a small but significant difference.^35^

We used a multi-beat approach to acquire 3D volumes. Multi-beat 3D images usually offer better temporal resolution than single-beat images, but may have lower spatial resolution due to stitching artifacts.^36^ However, the correlation with volumes measured by cardiac resonance was described to be excellent, regardless of the number of cardiac cycles used.^37^

Recently, new dedicated software to measure 3D LA volumes was developed^38^ and may result in slightly different data than ours. However, we described good inter- and intra-observer agreements for our 3D LA volume measurements^5^ and the correlation between non-dedicated 3D software to measure LA volume and cardiac resonance was described as significant.^39^

Our paper did not include enough individuals from all ethnic groups that compose the Brazilian population, such as Asian-Brazilians and native Indians. Another limitation is that we did not perform lab work to exclude patients who could be unaware of associated comorbidities. Also, we did not exclude patients who were overweight. In fact, 41 patients (53%) were overweight (BMI ≥ 25 and < 30 kg/m^2^). However, overweight is becoming increasingly prevalent among Brazilians and the total percentage in this article is close to the value disclosed by IBGE (56.9%).^40^

## Conclusions

Values for new echocardiographic indexes in normal Brazilian volunteers and their differences between men and women as well as their correlation with age are presented. However, we found differences in reference values between our study and others, which may be due to heterogeneity introduced by different machine vendors, age, gender distribution, ethnicity and the technique used to measure LV, LA or RV e.
